# Impact of abortion law reforms on women’s health services and outcomes: a systematic review protocol

**DOI:** 10.1186/s13643-021-01739-w

**Published:** 2021-06-28

**Authors:** Foluso Ishola, U. Vivian Ukah, Arijit Nandi

**Affiliations:** Department of Epidemiology, Biostatistics and Occupational Health, Faculty of Medicine, McGill University, Purvis Hall 1020 Pine Avenue West, Montreal, Quebec H3A 1A2 Canada

**Keywords:** Abortion law/policies; Impact, Unsafe abortion, Contraception, Fertility

## Abstract

**Background:**

A country’s abortion law is a key component in determining the enabling environment for safe abortion. While restrictive abortion laws still prevail in most low- and middle-income countries (LMICs), many countries have reformed their abortion laws, with the majority of them moving away from an absolute ban. However, the implications of these reforms on women’s access to and use of health services, as well as their health outcomes, is uncertain. First, there are methodological challenges to the evaluation of abortion laws, since these changes are not exogenous. Second, extant evaluations may be limited in terms of their generalizability, given variation in reforms across the abortion legality spectrum and differences in levels of implementation and enforcement cross-nationally. This systematic review aims to address this gap. Our aim is to systematically collect, evaluate, and synthesize empirical research evidence concerning the impact of abortion law reforms on women’s health services and outcomes in LMICs.

**Methods:**

We will conduct a systematic review of the peer-reviewed literature on changes in abortion laws and women’s health services and outcomes in LMICs. We will search Medline, Embase, CINAHL, and Web of Science databases, as well as grey literature and reference lists of included studies for further relevant literature. As our goal is to draw inference on the impact of abortion law reforms, we will include quasi-experimental studies examining the impact of change in abortion laws on at least one of our outcomes of interest. We will assess the methodological quality of studies using the quasi-experimental study designs series checklist. Due to anticipated heterogeneity in policy changes, outcomes, and study designs, we will synthesize results through a narrative description.

**Discussion:**

This review will systematically appraise and synthesize the research evidence on the impact of abortion law reforms on women’s health services and outcomes in LMICs. We will examine the effect of legislative reforms and investigate the conditions that might contribute to heterogeneous effects, including whether specific groups of women are differentially affected by abortion law reforms. We will discuss gaps and future directions for research. Findings from this review could provide evidence on emerging strategies to influence policy reforms, implement abortion services and scale up accessibility.

**Systematic review registration:**

PROSPERO CRD42019126927

**Supplementary Information:**

The online version contains supplementary material available at 10.1186/s13643-021-01739-w.

## Background

An estimated 25·1 million unsafe abortions occur each year, with 97% of these in developing countries [1–3]. Despite its frequency, unsafe abortion remains a major global public health challenge [4, 5]. According to the World health Organization (WHO), nearly 8% of maternal deaths were attributed to unsafe abortion, with the majority of these occurring in developing countries [5, 6]. Approximately 7 million women are admitted to hospitals every year due to complications from unsafe abortion such as hemorrhage, infections, septic shock, uterine and intestinal perforation, and peritonitis [7–9]. These often result in long-term effects such as infertility and chronic reproductive tract infections. The annual cost of treating major complications from unsafe abortion is estimated at US$ 232 million each year in developing countries [10, 11]. The negative consequences on children’s health, well-being, and development have also been documented. Unsafe abortion increases risk of poor birth outcomes, neonatal and infant mortality [12, 13]. Additionally, women who lack access to safe and legal abortion are often forced to continue with unwanted pregnancies, and may not seek prenatal care [14], which might increase risks of child morbidity and mortality.

Access to safe abortion services is often limited due to a wide range of barriers. Collectively, these barriers contribute to the staggering number of deaths and disabilities seen annually as a result of unsafe abortion, which are disproportionately felt in developing countries [15–17]. A recent systematic review on the barriers to abortion access in low- and middle-income countries (LMICs) implicated the following factors: restrictive abortion laws, lack of knowledge about abortion law or locations that provide abortion, high cost of services, judgmental provider attitudes, scarcity of facilities and medical equipment, poor training and shortage of staff, stigma on social and religious grounds, and lack of decision making power [17].

An important factor regulating access to abortion is abortion law [17–19]. Although abortion is a medical procedure, its legal status in many countries has been incorporated in penal codes which specify grounds in which abortion is permitted. These include prohibition in all circumstances, to save the woman’s life, to preserve the woman’s health, in cases of rape, incest, fetal impairment, for economic or social reasons, and on request with no requirement for justification [18–20].

Although abortion laws in different countries are usually compared based on the grounds under which legal abortions are allowed, these comparisons rarely take into account components of the legal framework that may have strongly restrictive implications, such as regulation of facilities that are authorized to provide abortions, mandatory waiting periods, reporting requirements in cases of rape, limited choice in terms of the method of abortion, and requirements for third-party authorizations [19, 21, 22]. For example, the Zambian Termination of Pregnancy Act permits abortion on socio-economic grounds. It is considered liberal, as it permits legal abortions for more indications than most countries in Sub-Saharan Africa; however, abortions must only be provided in registered hospitals, and three medical doctors—one of whom must be a specialist—must provide signatures to allow the procedure to take place [22]. Given the critical shortage of doctors in Zambia [23], this is in fact a major restriction that is only captured by a thorough analysis of the conditions under which abortion services are provided.

Additionally, abortion laws may exist outside the penal codes in some countries, where they are supplemented by health legislation and regulations such as public health statutes, reproductive health acts, court decisions, medical ethic codes, practice guidelines, and general health acts [18, 19, 24]. The diversity of regulatory documents may lead to conflicting directives about the grounds under which abortion is lawful [19]. For example, in Kenya and Uganda, standards and guidelines on the reduction of morbidity and mortality due to unsafe abortion supported by the constitution was contradictory to the penal code, leaving room for an ambiguous interpretation of the legal environment [25].

Regulations restricting the range of abortion methods from which women can choose, including medication abortion in particular, may also affect abortion access [26, 27]. A literature review contextualizing medication abortion in seven African countries reported that incidence of medication abortion is low despite being a safe, effective, and low-cost abortion method, likely due to legal restrictions on access to the medications [27].

Over the past two decades, many LMICs have reformed their abortion laws [3, 28]. Most have expanded the grounds on which abortion may be performed legally, while very few have restricted access. Countries like Uruguay, South Africa, and Portugal have amended their laws to allow abortion on request in the first trimester of pregnancy [29, 30]. Conversely, in Nicaragua, a law to ban all abortion without any exception was introduced in 2006 [31].

Progressive reforms are expected to lead to improvements in women’s access to safe abortion and health outcomes, including reductions in the death and disabilities that accompany unsafe abortion, and reductions in stigma over the longer term [17, 29, 32]. However, abortion law reforms may yield different outcomes even in countries that experience similar reforms, as the legislative processes that are associated with changing abortion laws take place in highly distinct political, economic, religious, and social contexts [28, 33]. This variation may contribute to abortion law reforms having different effects with respect to the health services and outcomes that they are hypothesized to influence [17, 29].

Extant empirical literature has examined changes in abortion-related morbidity and mortality, contraceptive usage, fertility, and other health-related outcomes following reforms to abortion laws [34–37]. For example, a study in Mexico reported that a policy that decriminalized and subsidized early-term elective abortion led to substantial reductions in maternal morbidity and that this was particularly strong among vulnerable populations such as young and socioeconomically disadvantaged women [38].

To the best of our knowledge, however, the growing literature on the impact of abortion law reforms on women’s health services and outcomes has not been systematically reviewed. A study by Benson et al. evaluated evidence on the impact of abortion policy reforms on maternal death in three countries, Romania, South Africa, and Bangladesh, where reforms were immediately followed by strategies to implement abortion services, scale up accessibility, and establish complementary reproductive and maternal health services [39]. The three countries highlighted in this paper provided unique insights into implementation and practical application following law reforms, in spite of limited resources. However, the review focused only on a selection of countries that have enacted similar reforms and it is unclear if its conclusions are more widely generalizable.

Accordingly, the primary objective of this review is to summarize studies that have estimated the causal effect of a change in abortion law on women’s health services and outcomes. Additionally, we aim to examine heterogeneity in the impacts of abortion reforms, including variation across specific population sub-groups and contexts (e.g., due to variations in the intensity of enforcement and service delivery). Through this review, we aim to offer a higher-level view of the impact of abortion law reforms in LMICs, beyond what can be gained from any individual study, and to thereby highlight patterns in the evidence across studies, gaps in current research, and to identify promising programs and strategies that could be adapted and applied more broadly to increase access to safe abortion services.

## Methods

The review protocol has been reported using Preferred Reporting Items for Systematic review and Meta-Analysis Protocols (PRISMA-P) guidelines [40] (Additional file 1). It was registered in the International Prospective Register of Systematic Reviews (PROSPERO) database CRD42019126927.

### Eligibility criteria

#### Types of studies

This review will consider quasi-experimental studies which aim to estimate the causal effect of a change in a specific law or reform and an outcome, but in which participants (in this case jurisdictions, whether countries, states/provinces, or smaller units) are not randomly assigned to treatment conditions [41]. Eligible designs include the following:

- Pretest-posttest designs where the outcome is compared before and after the reform, as well as nonequivalent groups designs, such as pretest-posttest design that includes a comparison group, also known as a controlled before and after (CBA) designs.
- Interrupted time series (ITS) designs where the trend of an outcome after an abortion law reform is compared to a counterfactual (i.e., trends in the outcome in the post-intervention period had the jurisdiction not enacted the reform) based on the pre-intervention trends and/or a control group [42, 43].
- Differences-in-differences (DD) designs, which compare the before vs. after change in an outcome in jurisdictions that experienced an abortion law reform to the corresponding change in the places that did not experience such a change, under the assumption of parallel trends [44, 45].
- Synthetic controls (SC) approaches, which use a weighted combination of control units that did not experience the intervention, selected to match the treated unit in its pre-intervention outcome trend, to proxy the counterfactual scenario [46, 47].
- Regression discontinuity (RD) designs, which in the case of eligibility for abortion services being determined by the value of a continuous random variable, such as age or income, would compare the distributions of post-intervention outcomes for those just above and below the threshold [48].

There is heterogeneity in the terminology and definitions used to describe quasi-experimental designs, but we will do our best to categorize studies into the above groups based on their designs, identification strategies, and assumptions.

Our focus is on quasi-experimental research because we are interested in studies evaluating the effect of population-level interventions (i.e., abortion law reform) with a design that permits inference regarding the causal effect of abortion legislation, which is not possible from other types of observational designs such as cross-sectional studies, cohort studies or case-control studies that lack an identification strategy for addressing sources of unmeasured confounding (e.g., secular trends in outcomes). We are not excluding randomized studies such as randomized controlled trials, cluster randomized trials, or stepped-wedge cluster-randomized trials; however, we do not expect to identify any relevant randomized studies given that abortion policy is unlikely to be randomly assigned. Since our objective is to provide a summary of empirical studies reporting primary research, reviews/meta-analyses, qualitative studies, editorials, letters, book reviews, correspondence, and case reports/studies will also be excluded.

### Population

Our population of interest includes women of reproductive age (15–49 years) residing in LMICs, as the policy exposure of interest applies primarily to women who have a demand for sexual and reproductive health services including abortion.

### Intervention

The intervention in this study refers to a change in abortion law or policy, either from a restrictive policy to a non-restrictive or less restrictive one, or vice versa. This can, for example, include a change from abortion prohibition in all circumstances to abortion permissible in other circumstances, such as to save the woman’s life, to preserve the woman’s health, in cases of rape, incest, fetal impairment, for economic or social reasons, or on request with no requirement for justification. It can also include the abolition of existing abortion policies or the introduction of new policies including those occurring outside the penal code, which also have legal standing, such as:

- National constitutions;
- Supreme court decisions, as well as higher court decisions;
- Customary or religious law, such as interpretations of Muslim law;
- Medical ethical codes; and
- Regulatory standards and guidelines governing the provision of abortion.

We will also consider national and sub-national reforms, although we anticipate that most reforms will operate at the national level.

### Comparator

The comparison group represents the counterfactual scenario, specifically the level and/or trend of a particular post-intervention outcome in the treated jurisdiction that experienced an abortion law reform had it, counter to the fact, not experienced this specific intervention. Comparison groups will vary depending on the type of quasi-experimental design. These may include outcome trends after abortion reform in the same country, as in the case of an interrupted time series design without a control group, or corresponding trends in countries that did not experience a change in abortion law, as in the case of the difference-in-differences design.

### Outcome measures

#### Primary outcomes

- Access to abortion services: There is no consensus on how to measure access but we will use the following indicators, based on the relevant literature [49]: [1] the availability of trained staff to provide care, [2] facilities are geographically accessible such as distance to providers, [3] essential equipment, supplies and medications, [4] services provided regardless of woman’s ability to pay, [5] all aspects of abortion care are explained to women, [6] whether staff offer respectful care, [7] if staff work to ensure privacy, [8] if high-quality, supportive counseling is provided, [9] if services are offered in a timely manner, and [10] if women have the opportunity to express concerns, ask questions, and receive answers.
- Use of abortion services refers to induced pregnancy termination, including medication abortion and number of women treated for abortion-related complications.

#### Secondary outcomes

- Current use of any method of contraception refers to women of reproductive age currently using any method contraceptive method.
- Future use of contraception refers to women of reproductive age who are not currently using contraception but intend to do so in the future.
- Demand for family planning refers to women of reproductive age who are currently using, or whose sexual partner is currently using, at least one contraceptive method.
- Unmet need for family planning refers to women of reproductive age who want to stop or delay childbearing but are not using any method of contraception.
- Fertility rate refers to the average number of children born to women of childbearing age.
- Neonatal morbidity and mortality refer to disability or death of newborn babies within the first 28 days of life.
- Maternal morbidity and mortality refer to disability or death due to complications from pregnancy or childbirth.

### Timing

There will be no language, date, or year restrictions on studies included in this systematic review.

### Setting

Studies have to be conducted in a low- and middle-income country. We will use the country classification specified in the World Bank Data Catalogue to identify LMICs (Additional file 2).

### Search methods

We will perform searches for eligible peer-reviewed studies in the following electronic databases.

- Ovid MEDLINE(R) (from 1946 to present)
- Embase Classic+Embase on OvidSP (from 1947 to present)
- CINAHL (1973 to present); and
- Web of Science (1900 to present)

The reference list of included studies will be hand searched for additional potentially relevant citations. Additionally, a grey literature search for reports or working papers will be done with the help of Google and Social Science Research Network (SSRN).

### Search strategy

A search strategy, based on the eligibility criteria and combining subject indexing terms (i.e., MeSH) and free-text search terms in the title and abstract fields, will be developed for each electronic database. The search strategy will combine terms related to the interventions of interest (i.e., abortion law/policy), etiology (i.e., impact/effect), and context (i.e., LMICs) and will be developed with the help of a subject matter librarian. We opted not to specify outcomes in the search strategy in order to maximize the sensitivity of our search. See Additional file 3 for a draft of our search strategy.

### Data collection and analysis

#### Data management

Search results from all databases will be imported into Endnote reference manager software (Version X9, Clarivate Analytics) where duplicate records will be identified and excluded using a systematic, rigorous, and reproducible method that utilizes a sequential combination of fields including author, year, title, journal, and pages. Rayyan systematic review software will be used to manage records throughout the review [50].

#### Selection process

Two review authors will screen titles and abstracts and apply the eligibility criteria to select studies for full-text review. Reference lists of any relevant articles identified will be screened to ensure no primary research studies are missed. Studies in a language different from English will be translated by collaborators who are fluent in the particular language. If no such expertise is identified, we will use Google Translate [51]. Full text versions of potentially relevant articles will be retrieved and assessed for inclusion based on study eligibility criteria. Discrepancies will be resolved by consensus or will involve a third reviewer as an arbitrator. The selection of studies, as well as reasons for exclusions of potentially eligible studies, will be described using a PRISMA flow chart.

#### Data extraction

Data extraction will be independently undertaken by two authors. At the conclusion of data extraction, these two authors will meet with the third author to resolve any discrepancies. A piloted standardized extraction form will be used to extract the following information: authors, date of publication, country of study, aim of study, policy reform year, type of policy reform, data source (surveys, medical records), years compared (before and after the reform), comparators (over time or between groups), participant characteristics (age, socioeconomic status), primary and secondary outcomes, evaluation design, methods used for statistical analysis (regression), estimates reported (means, rates, proportion), information to assess risk of bias (sensitivity analyses), sources of funding, and any potential conflicts of interest.

### Risk of bias and quality assessment

Two independent reviewers with content and methodological expertise in methods for policy evaluation will assess the methodological quality of included studies using the quasi-experimental study designs series risk of bias checklist [52]. This checklist provides a list of criteria for grading the quality of quasi-experimental studies that relate directly to the intrinsic strength of the studies in inferring causality. These include [1] relevant comparison, [2] number of times outcome assessments were available, [3] intervention effect estimated by changes over time for the same or different groups, [4] control of confounding, [5] how groups of individuals or clusters were formed (time or location differences), and [6] assessment of outcome variables. Each of the following domains will be assigned a “yes,” “no,” or “possibly” bias classification. Any discrepancies will be resolved by consensus or a third reviewer with expertise in review methodology if required.

### Confidence in cumulative evidence

The strength of the body of evidence will be assessed using the Grades of Recommendation, Assessment, Development and Evaluation (GRADE) system [53].

### Data synthesis

We anticipate that risk of bias and heterogeneity in the studies included may preclude the use of meta-analyses to describe pooled effects. This may necessitate the presentation of our main findings through a narrative description. We will synthesize the findings from the included articles according to the following key headings:

- Information on the differential aspects of the abortion policy reforms.
- Information on the types of study design used to assess the impact of policy reforms.
- Information on main effects of abortion law reforms on primary and secondary outcomes of interest.
- Information on heterogeneity in the results that might be due to differences in study designs, individual-level characteristics, and contextual factors.

#### Potential meta-analysis

If outcomes are reported consistently across studies, we will construct forest plots and synthesize effect estimates using meta-analysis. Statistical heterogeneity will be assessed using the I^2^ test where I^2^ values over 50% indicate moderate to high heterogeneity [54]. If studies are sufficiently homogenous, we will use fixed effects. However, if there is evidence of heterogeneity, a random effects model will be adopted. Summary measures, including risk ratios or differences or prevalence ratios or differences will be calculated, along with 95% confidence intervals (CI).

#### Analysis of subgroups

If there are sufficient numbers of included studies, we will perform sub-group analyses according to type of policy reform, geographical location and type of participant characteristics such as age groups, socioeconomic status, urban/rural status, education, or marital status to examine the evidence for heterogeneous effects of abortion laws.

#### Sensitivity analysis

Sensitivity analyses will be conducted if there are major differences in quality of the included articles to explore the influence of risk of bias on effect estimates.

### Meta-biases

If available, studies will be compared to protocols and registers to identify potential reporting bias within studies. If appropriate and there are a sufficient number of studies included, funnel plots will be generated to determine potential publication bias.

## Discussion

This systematic review will synthesize current evidence on the impact of abortion law reforms on women’s health. It aims to identify which legislative reforms are effective, for which population sub-groups, and under which conditions.

Potential limitations may include the low quality of included studies as a result of suboptimal study design, invalid assumptions, lack of sensitivity analysis, imprecision of estimates, variability in results, missing data, and poor outcome measurements. Our review may also include a limited number of articles because we opted to focus on evidence from quasi-experimental study design due to the causal nature of the research question under review. Nonetheless, we will synthesize the literature, provide a critical evaluation of the quality of the evidence and discuss the potential effects of any limitations to our overall conclusions. Protocol amendments will be recorded and dated using the registration for this review on PROSPERO. We will also describe any amendments in our final manuscript.

Synthesizing available evidence on the impact of abortion law reforms represents an important step towards building our knowledge base regarding how abortion law reforms affect women’s health services and health outcomes; we will provide evidence on emerging strategies to influence policy reforms, implement abortion services, and scale up accessibility. This review will be of interest to service providers, policy makers and researchers seeking to improve women’s access to safe abortion around the world.

## Supplementary Information

**Additional File 1: **PRISMA-P 2015 Checklist. This checklist has been adapted for use with systematic review protocol submissions to BioMed Central journals from Table 3 in Moher D et al: Preferred reporting items for systematic review and meta-analysis protocols (PRISMA-P) 2015 statement. *Systematic Reviews* 2015 4:1

**Additional File 2:.** LMICs according to World Bank Data Catalogue. Country classification specified in the World Bank Data Catalogue to identify low- and middle-income countries

**Additional File 3: Table 1**. Search strategy in Embase. Detailed search terms and filters applied to generate our search in Embase

## Abbreviations

CINAHL: Cumulative index to nursing and allied health literature
EMBASE: Excerpta medica database
LMICs: Low- and middle-income countries
PRISMA-P: Preferred reporting items for systematic review and meta-analysis protocols
PROSPERO: International prospective register of systematic reviews
