# Supplementary material for: Impact of abortion law reforms on women’s health services and outcomes: a systematic review protocol
Source: Syst Rev. 2021 Jun 28;10:192. doi: 10.1186/s13643-021-01739-w (PMC8240208; doi:10.1186/s13643-021-01739-w)
Supplement: Supplementary file 2 — Additional File 2:. LMICs according to World Bank Data Catalogue. Country classification specified in the World Bank Data Catalogue to identify low- and middle-income countries [file 13643_2021_1739_MOESM2_ESM.docx]

Additional File 2

LMICs according to World Bank Data Catalogue

| LOW- & MIDDLE-INCOME COUNTRIES | | | | | |
| --- | --- | --- | --- | --- | --- |
| AFGHANISTAN | CHINA | GUINEA | MALDIVES | ROMANIA | TURKEY |
| ALBANIA | COLOMBIA | GUINEA-BISSAU | MALI | RUSSIAN FEDERATION | TURKMENISTAN |
| ALGERIA | COMOROS | GUYANA | MARSHALL ISLANDS | RWANDA | TUVALU |
| AMERICAN SAMOA | CONGO, DEM. REP. | HAITI | MAURITANIA | SAMOA | UGANDA |
| ANGOLA | CONGO, REP. | HONDURAS | MAURITIUS | SAO TOME AND PRINCIPE | UKRAINE |
| ARGENTINA | COSTA RICA | INDIA | MEXICO | SENEGAL | UZBEKISTAN |
| ARMENIA | COTE D’IVOIRE | INDONESIA | MICRONESIA, FED.STS. | SERBIA | VANUATU |
| AZERBAIJAN | CUBA | IRAN, ISLAMIC REP. | MOLDOVA | SIERRA LEONE | VENEZUELA, RB |
| BANGLADESH | DJIBOUTI | IRAQ | MONGOLIA | SOLOMON ISLANDS | VIETNAM |
| BELARUS | DOMINICA | JAMAICA | MONTENEGRO | SOMALIA | WEST BANK AND GAZA |
| BELIZE | DOMINICAN REPUBLIC | JORDAN | MOROCCO | SOUTH AFRICA | YEMEN, REP. |
| BENIN | ECUADOR | KAZAKHSTAN | MOZAMBIQUE | SOUTH SUDAN | ZAMBIA |
| BHUTAN | EGYPT, ARAB REP. | KENYA | MYANMAR | SRI LANKA | ZIMBABWE |
| BOLIVIA | EL SALVADOR | KIRIBATI | NAMIBIA | ST. LUCIA |  |
| BOSNIA AND HERZEGOVINA | EQUATORIAL GUINEA | KOREA, DEM. PEOPLE’S REP. | NAURU | ST. VINCENT AND THE GRENADINES |  |
| BOTSWANA | ERITREA | KOSOVO | NEPAL | SUDAN |  |
| BRAZIL | ESWATINI | KYRGYZ REPUBLIC | NICARAGUA | SURINAME |  |
| BULGARIA | ETHIOPIA | LAO PDR | NIGER | SYRIAN ARAB REPUBLIC |  |
| BURKINA FASO | FIJI | LEBANON | NIGERIA | TAJIKISTAN |  |
| BURUNDI | GABON | LESOTHO | NORTH MACEDONIA | TANZANIA |  |
| CABO VERDE | GAMBIA, THE | LIBERIA | PAKISTAN | THAILAND |  |
| CAMBODIA | GEORGIA | LIBYA | PAPUA NEW GUINEA | TIMOR-LESTE |  |
| CAMEROON | GHANA | MADAGASCAR | PARAGUAY | TOGO |  |
| CENTRAL AFRICAN REPUBLIC | GRENADA | MALAWI | PERU | TONGA |  |
| CHAD | GUATEMALA | MALAYSIA | PHILLIPINES | TUNISIA |  |
